# The efficacy of iron chelator regimes in reducing cardiac and hepatic iron in patients with thalassaemia major: a clinical observational study

**DOI:** 10.1186/1532-429X-11-20

**Published:** 2009-06-28

**Authors:** Vasilios Berdoukas, Giorgos Chouliaras, Panagiotis Moraitis, Kirykos Zannikos, Eleni Berdoussi, Vassilios Ladis

**Affiliations:** 1 Thalassaemia Unit, First Department of Paediatrics, University of Athens, "Aghia Sophia" Children's Hospital, Athens, Greece

## Abstract

**Background:**

Available iron chelation regimes in thalassaemia may achieve different changes in cardiac and hepatic iron as assessed by MR. The aim of this study was to assess the efficacy of four available iron chelator regimes in 232 thalassaemia major patients by assessing the rate of change in repeated measurements of cardiac and hepatic MR.

**Results:**

For the heart, deferiprone and the combination of deferiprone and deferoxamine significantly reduced cardiac iron at all levels of iron loading. As patients were on deferasirox for a shorter time, a second analysis ("Initial interval analysis") assessing the change between the first two recorded MR results for both cardiac and hepatic iron (minimum interval 12 months) was made. Combination therapy achieved the most rapid fall in cardiac iron load at all levels and deferiprone alone was significantly effective with moderate and mild iron load. In the liver, deferasirox effected significant falls in iron load and combination therapy resulted in the most rapid decline.

**Conclusion:**

With the knowledge of the efficacy of the different available regimes and the specific iron load in the heart and the liver, appropriate tailoring of chelation therapy should allow clearance of iron. Combination therapy is best in reducing both cardiac and hepatic iron, while monotherapy with deferiprone or deferasirox are effective in the heart and liver respectively. The outcomes of this study may be useful to physicians as to the chelation they should prescribe according to the levels of iron load found in the heart and liver by MR.

## Background

In beta thalassaemia major patients (TM) transfusions and iron chelation therapy have significantly improved the survival and reduced morbidity [1-4]. However, heart complications still represent significant morbidity and remain the leading cause of mortality [2]. In some cases this was because of the difficulty in accepting the chelation treatment with deferoxamine, which was cumbersome [5], but problems occurred even in some patients who accepted the chelation therapy well [6]. Surrogate markers such as ferritin levels and liver iron concentration (LIC), though correlated to the incidence of cardiac disease, did not have predictive value with respect to cardiac function [7] nor to the degree of cardiac iron load [8-10]. Liver iron reflects the total body iron load [11], and because hepatic complications are the third most common cause of death and iron overload plays a role with respect to the incidence of hepatic carcinoma [12], knowledge of the degree of iron loading in both heart and liver through non-invasive imaging is essential. The availability of Magnetic Resonance Imaging (MR) [13] allows indirect assessment of cardiac and hepatic iron. With the availability of three iron chelators, deferoxamine (DFO), deferiprone (DFP) and deferasirox (DFX) together with the combination of DFO and DFP (Comb), we sought to assess the efficacy of the available regimes on improving cardiac and hepatic iron load as measured by T2* MR imaging. To date there are some prospective studies that have assessed the efficacy of the chelators, either as monotherapy or in combination. In this observational study, we analysed the efficacy of the four currently available chelation regimes in a large clinic setting.

## Methods

### Patients

Patients attending our centre are usually transfused at bi-weekly intervals maintaining a mean pre-transfusion haemoglobin level above 95 g/l. Chelation regimes include DFO since the mid 1970's, DFP from 2000, combinations of the two since 2002 [14] and after 2007 monotherapy with DFX. All the patients on DFO were prescribed between 30–45 mg/kg/infusion 5–7 days per week, those on DFP between 75–100 mg/kg/day and those on DFX between 15–40 mg/kg/day. Only two patients on DFX were receiving 15 mg/kg/day and the mean dose for all patients was 26.6 mg/kg/day. The doses for combination therapy were similar to monotherapy however the days of DFO treatment were variable with a minimum of 3 days per week. Since MR for the assessment of cardiac and hepatic load became available, the patients were referred for such studies at variable intervals described in the results. The recommended regime, doses of the individual chelators and the frequency of DFO infusions were initially changed or adjusted according to ferritin levels and subsequently were based on clinical features – particularly ferritin levels and the MR findings, side effects, and patient request. These changes were made at any time and not necessarily related to the timing of the MR scans.

The Athens MR Imaging site (Euromedica Encephalos) was validated by the Royal Brompton Hospital for T2* [15]. A cardiac-dedicated General Electric 1.5 Tesla magnet (Signa CVI with 40 mT/m gradients and appropriate cardiac software) was used for the MR measurements. For cardiac T2* determination a single breath multi-echo fast gradient-echo sequence was used with a fixed TR of 25.6 ms, 10 echoes acquired in the range of 2.2–22.6 ms and an inter-echo spacing of about 2.2 ms). Additional imaging parameters were 1 excitation, matrix of 160 × 256, bandwidth 62.5 KHz, slice thickness of 8 mm, ECG-gating and total acquisition time ranging from 15–20 msec. Normal values for myocardial T2* ranged between 25 to 47.6 ms (mean = 33.8 ms^-1^). The calculation of T2* was made off line by an exponential least-squares fit of the multi-echo signal intensity data (SI) versus echo time (TE), with the use of a PC and standard fitting programs (GRAFIT or SigmaPlot 2001).

Transfusion dependent patients with more than one MR assessment were included in the analysis. At the time that MR T2* for iron estimation became available, hepatic T2* was not always performed. For this reason there are fewer hepatic observations than cardiac.

The change in cardiac T2* was assessed according to the chelation regime the patient was receiving during the period spanning the MR scan. Due to the variable time intervals between MR scans, the results are expressed as mean annual change. Participants switched treatment groups during the study period, therefore contributing information to more than one regime. Only studies on patients who were on a particular regime for more than 50% of the time interval between MR scans and the exposure during that time to the particular regime was greater than 6 months, were included in the assessment.

Equal changes in T2* correspond to disproportionate changes in tissue iron concentration depending on the starting T2* level [16,17]. For example an increase of 3 ms in T2* from 5 ms to 8 ms reflects an estimated decrease in cardiac iron of 2 mg/g. dry weight (mg/g dw) while an equal increase in T2* from 17 ms to 20 ms corresponds to a fall of 0.24 mg/g dw. To demonstrate changes more quantitatively and descriptively, additional analyses were performed according to cardiac and hepatic iron concentration using an estimate of the conversion of T2* and its changes, to mg/g dw. For the heart, we converted the T2* to Cardiac Iron Concentration (CIC) using the formula adapted from Wood *et al *[16], (1/T2*-1/T2_0 _*)/K_dry _where T2_0 _* is 33.8 ms (our laboratory's T2_0 _*) and the slope, K_dry_, has been estimated at 37.4 Hz·mg^-1^·g dry weight^-1^. For further clarification in the figures, we also show the changes in R2* (1000/T2*) as it also has a linear relationship to the cardiac iron and is more interpretable quantitatively than T2*. The heavily iron loaded group included patients with T2* < 8 ms (>2.6 mg/g dw, R2* > 125 sec^-1^), moderate ≥ 8 and < 14 (1.1–2.6 mg/g dw, R2* 71.4 – 125 sec^-1^) and mild ≥ 14 and < 20 ms (0.6–1.1 mg/g dw, R2* 50–71.4 sec^-1^). Acceptable levels were those ≥ 20 ms (<0.6 mg/g dw, R2* ≤ 50 sec^-1^). Although prognostic cut offs for T2* have not yet been clearly established, the abovementioned classification is widely used in clinical practice. For the liver, we used the conversion from T2* to LIC, by using the formula, 0.202+0.0254xR2* adapted from Wood *et al*. [17] and is also the calculation used by our MR service. The heavily iron loaded group included patients with T2* < 1.6 ms (16.1 mg/g dw), moderate ≥ 1.6 and < 4.0 (6.6–16.1 mg/g dw) and mild ≥ 4 and < 9 ms (3–6.6 mg/g dw). Acceptable levels were those ≥ 9 ms (<3 mg/g dw) [17,18].

We also determined whether any patients who were above the acceptable levels (Cardiac T2* ≥ 20 ms and hepatic T2* ≥ 9.0 ms) fell below those levels.

Clinical practice differs from designed trials in that many parameters cannot be controlled. All assessments were therefore made according to the Intention to Treat i.e. compliance with chelation therapy was not assessed or taken into account for the purposes of this study, time intervals between MR scans were not consistent and management changes were often related to the degree of perceived iron load. In addition, dose responses were not analysed.

The Ethics Committee of the hospital approved permission for medical review, waiver of informed consent and anonymous publication of data according to the Declaration of Helsinki.

### Statistical analysis

Data on each patient were retrospectively recorded from the patient histories. The effect of the chelation regime in reducing iron was then analysed longitudinally. Continuous variables are presented as mean ± SD while categorical variables are described using absolute and relative frequencies. Means were compared by Student's t-test. Wherever sample sizes were less than 30 or in cases of extremely skewed distributions, Wilcoxon's matched-pairs signed-ranks or the Mann-Whitney test were used. Hypotheses on whether the mean of a continuous variable differed significantly between more than two groups were assessed by analysis of variance (ANOVA). Alternatively, the Kruskal Wallis test was used when the sample sizes were small. Multiple hypothesis testing was corrected by the Bonferroni method. Categorical variables were tested using Fisher's exact test. Wherever descriptive data appear for each treatment group, it refers to the patients who had at least two MR scans when exposed to a particular regime. In this way a patient may contribute information to more than one group. For example, a patient, who has had two MR scans and has been on DFO during that period and then switched to Comb with a subsequent MR, will be included in the DFO group once and in the Comb group once. This patient will then contribute information regarding MR changes and descriptive statistics such as age to both groups.

The statistical method described below depends on the normality of the data to be analyzed. For this reason, we chose to analyse T2* values because these appear to have the least deviation from the Normal distribution compared to R2* (1000/T2*), CIC, LIC or other log transformations of T2* values and have a rather more symmetric distribution than any of those (analyses available from authors). After extraction of the results, we transformed the change in T2* to mg/gdw, using the above mentioned equation from data from Wood et al [16] in order to eliminate the lack of linearity between tissue iron and T2*, accepting that at this time, no definite calibration curve between T2* and cardiac iron for humans has been established. The described approach fulfils the necessary statistical assumptions and translates the results to tissue iron concentration which is more relevant and interpretable to clinicians than a number value (in ms or sec^-1^).

In order to evaluate the rate of change of T2* (r-T2*), a repeated measurements regression analysis with correlated errors was applied. Our data set consisted of 6 observations per person (maximum number of MR scans in our population). For those who had less than 6 MR scans the remaining values were considered missing. Our data were imbalanced because of the varying time intervals between MR scans. For example the second MR for one individual might have been at 12 months and for another it may have been at 20 months. In the same way the third MR could have been at no fixed time after the second. For this reason we incorporated time in a continuous manner (and not as pre-fixed categorical time point) using four different time covariates that measured time of exposure for each treatment group, allowing for a direct estimation of the effect of each regime on the rate of change of T2* (r-T2*). Whenever a patient switched to a different regime the respective time covariate between MR scans under the specific medication began to be measured. Assuming that the rate of change of T2* (r-T2*) depends on the level of siderosis we used a time-varying categorical covariate (four levels of siderosis for heart and liver as described earlier) to adjust for the previous (baseline) measurement of T2*. Furthermore, in order to quantify the rates of change of T2* for each regime within each level of siderosis we used four interaction terms between the 4 time variables and the time-varying baseline. Finally the potential effect of gender and age was also assessed with the latter being incorporated in the model in a time-varying manner. Due to the relatively large sample size an unstructured covariance matrix describing the correlation of the repeated measurements within the same person was used. The significance of the effect of each term was evaluated by the Wald test.

In the "Initial interval analysis", we only analyzed the first two MR scans with a minimal interval of 12 months on each regime. Initially we assessed the response (change in cardiac iron – Δ CIC and the change in hepatic iron – Δ LIC) to each regime. A comparison of Δ CIC or Δ LIC between the four regimes without taking time into account would be inappropriate. In order to evaluate the differences of the rates of change of CIC and LIC between the four treatment regimes, subsequently, annual rates were calculated (annual rate: r-CIC = Δ CIC/time and r-LIC = Δ LIC/time with the time interval measured in years). All statistical procedures were performed using SAS V8 statistical software (SAS Institute Inc., Cary, NC, USA) and Stata 8.0 (StataCorp, College Station, Texas.USA).

## Results

### General

Two hundred and thirty two patients had more than one MR with a total of 652 studies. 83% of patients had 2 or 3 studies while 17% had 4 to 6.

Mean baseline ages (years) did not differ significantly between treatment groups (DFO 29.4, DFP 29.8, Comb 28.9 and DFX 27.5 years, ANOVA F test p = 0.17) the male female proportions differed significantly in that more women were on DFX than men. Table 1 shows the mean baseline cardiac and hepatic T2* values for each group and the time of exposure to each chelation regime between MR scans.

**Table 1:** Baseline T2* values and period of exposure to each chelation regime between MR scans.

Parameter, mean (SD)	Treatment				
	
	DFO	DFP	DFP & DFO	DFX	ANOVA F-test p-value
Heart T2*	21.4 (12.6)	15.7 (9.6)	16.6 (10.8)	27 (9.5)	<0.0001
Liver T2*	6.2 (5.5)	10.6 (9.3)	5 (5.3)	5.3 (6.8)	0.0004
Exposure period (months)	24 (11.5)	25.6 (13.2)	26.8 (12.6)	17.3 (7)	<0.0001
Intervals between MR scans (months)	19.1 (9.6)	17.2 (11.3)	19 (9.5)	14 (4.5)	0.0001

In both types of analysis, patients whose baseline levels were acceptable (Cardiac T2* ≥ 20 ms, Hepatic T2* ≥ 9 ms) were analyzed but are not shown, as their results were not of clinical significance.

### Cardiac results

**Figure 1:**
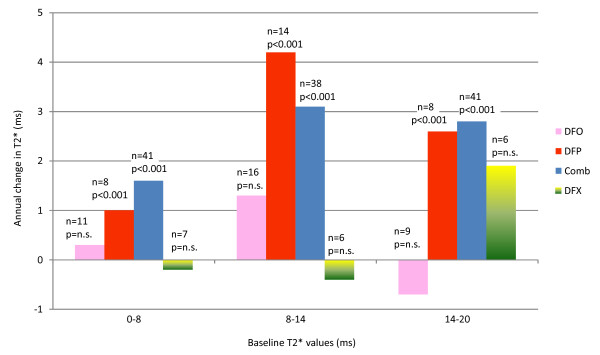
Rate of change of T2* according to severity of baseline cardiac iron load and chelation regime prescribed.

The hypothesis that the rate of change of T2* depends on baseline iron level was confirmed by the repeated measurements analysis. The interaction term between the rate of change in T2* and the baseline level of siderosis appears significant in the case of DFO, DFP and Comb, but not in the case of DFX. Based on these results all the comparisons are reported before and after stratification. The estimated annual rates of change in T2*(r-T2*) for each regime according to the severity of the cardiac iron loading (results adjusted for age – gender was not significant), are presented in figure 1. Only DFP or Comb demonstrated significant improvement in cardiac T2* at all levels of cardiac iron loading.

**Figure 2:**
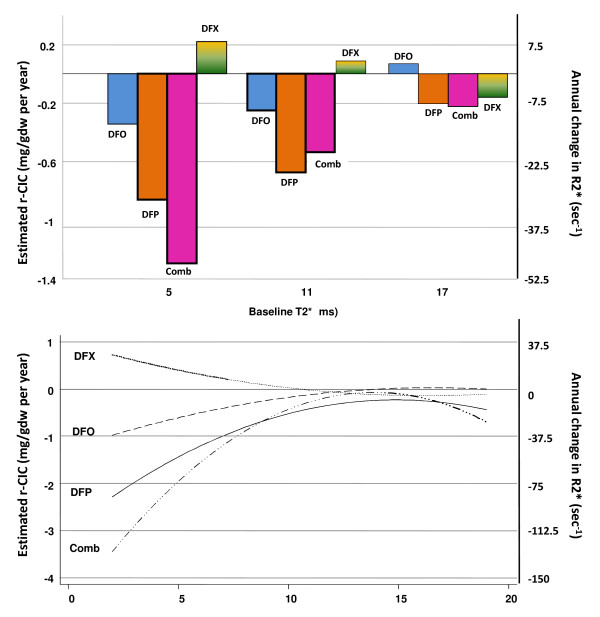
Estimated annual rates of change of CIC and R2*. Estimated annual rates of change of CIC (r-CIC, mg/gdw per year) and R2* (sec-1) for each regime at starting T2* levels of 5, 11 and 17 ms (top panel) and quadratic fit curves respectively (bottom panel) as evaluated from the "repeated measurements analysis".

Due to the non-linear relationship between T2* and CIC, the estimated constant rate of change of T2* within each severity group corresponds to a variable rate of change for CIC within the same severity group. For this reason, we produced estimates of the annual change of CIC for baseline measurements of 5, 11 and 17 ms (Figure 2, top panel). These values represent, more or less, the middle of each severity range. A quadratic fit curve based on many estimations of r-CIC according to a wide range of values of the baseline T2* (2–18 ms) is also presented in Figure 2 for each treatment regime (bottom panel). The observed increasing trend for r-CIC for baseline T2* values > 15 ms is due to the discontinuous form of iron severity grouping which produced overlapping of the estimated r-CIC between the three iron load groups. This would have been less likely to occur if an extremely large number of patients was available, allowing for a very narrow categorization.

**Figure 3:**
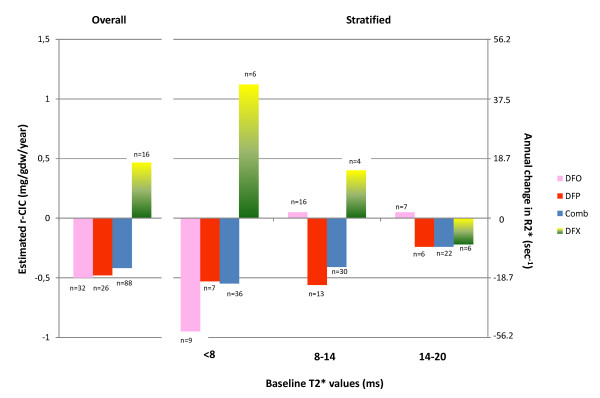
**Annual rate of change of Estimated Cardiac Iron Concentration r-CIC comparing significance between the treatment regimes (Initial interval analysis)**. Significant comparisons (Mann Witney Test) • Overall: DFP -v- DFX p < 0.001, Comb -v- DFX p < 0.001 • Severe: Comb -v- DFX p = 0.04, DFX -v- DFO p = 0.07 (marginally non-significant) • Moderate: DFP -v- DFO p = 0.06, DFP -v- DFX p = 0.036, Comb -v- DFO p = 0.07, Comb -v- DFX p = 0.07 (both marginally non-significant) • Mild: No comparison was statistically significant.

In the "Initial interval analysis", Wilcoxon signed-rank test showed a significant reduction of CIC (ΔCIC, mg/gdw) in the DFP and the Comb groups, but not in the DFO and the DFX groups. Due to the variability in the rate of change in T2* seen in the repeated measurement analysis, we performed the analysis after stratifying for the baseline iron load level (Table 2). In this analysis, Comb was effective at all levels of iron load, DFP at moderate and mild levels and DFO in the heavy loaded patients. Due to the significant difference in the time periods of exposure to the chelation regime, the annual rate of change of T2* in the "Initial interval analysis" were analysed and compared. These results are presented in figure 3.

**Table 2:** Mean estimated^† ^change in CIC mg/gdw (Δ CIC) according to severity of cardiac siderosis between first two MR scans ("Initial interval analysis" -minimum interval 12 months).

		Overall			Stratified								
					
					Heavy			Moderate			Mild		
Regime	Time* (years)	n	Δ CIC	p	n	Δ CIC	p	n	Δ CIC	p	n	Δ CIC	p

DFO	1.83	32	-0.23	0.43	9	-1.3	0.028	16	0.16	0.71	7	+0.27	0.95

DFP	1.80	26	-0.64	0.001	7	-0.57	0.49	13	-0.82	0.002	6	-0.36	0.027

Comb	1.80	88	-0.78	<0.001	36	-1.12	0.002	30	-0.69	<0.001	22	-0.41	<0.001

DFX	1.33	16	+0.66	0.10	6	+1.43	0.24	4	+0.73	0.46	6	-0.15	0.17

*Time = mean time (in years) between MR scans.

^†^Wilcoxon matched-pairs signed rank test

Of the patients who had T2* ≥ 20 ms only one demonstrated a fall in cardiac T2* to less than 20 ms over the first interval. This patient was on Comb.

### Hepatic results

The "Initial interval analysis" (Wilcoxon signed-rank test) showed a significant reduction of LIC in the Comb and the DFX groups, but not in the DFP or the DFO groups (Table 3). The Δ LIC was not constant across the three levels of liver iron load for each regime. The results of the stratified analysis are shown in table 3. Combination therapy caused a significant reduction in LIC at all levels of haemosiderosis while DFX reduced the LIC in severely and moderately loaded patients.

**Table 3:** Mean estimated^† ^change in LIC mg/gdw (Δ LIC) according to severity of hepatic siderosis between first two MR scans (minimum interval 12 months).

		Overall			Stratified								
					
					Heavy			Moderate			Mild		
Regime	Time* (years)	n	Δ LIC	p	n	Δ LIC	p	n	Δ LIC	p	n	Δ LIC	p

DFO	2.0	36	+1.34	0.095	5	+2.31	0.68	11	+1.64	0.21	20	+0.94	0.27

DFP	1.9	14	-6.2	0.068	3	n.a.^∂^	n.a.	6	-0.97	0.3	5	+2.68	0.5

Comb	1.8	99	-4.19	<0.001	25	-9.18	0.003	45	-3.27	<0.001	29	-1.33	0.004

DFX	1.3	53	-2.80	0.005	21	-5.38	0.042	16	-2.08	0.042	16	-0.14	0.35

*Time = mean time (in years) between MR scans.

^†^Wilcoxon matched-pairs signed rank test

^∂ ^Numbers too small for analysis

We analyzed the estimated mean difference of annual rate of change of LIC (r-LIC) for all four regimes using the same approach as for the heart. Non-stratified analysis showed significant differences in r-LIC among the four treatments (DFO: r-LIC = 0.50 (increase) mg/g dw, n = 36; DFP: r-LIC = -3.69 mg/g dw, n = 14; Comb: r-LIC = -3.14 mg/g dw, n = 99; DFX: r-LIC = -2.15 mg/g dw, n = 53; Kruskal Wallis test: p < 0.001). Significant comparisons (Mann-Whitney test) were between Comb and DFO (p < 0.001), and between DFX and DFO (p = 0.02) while between DFP and DFO the result was marginally non-significant (p = 0.066). The analysis of r-LIC was repeated after stratification revealing the following significant results:

• In the moderate group, the annual rate of change in LIC was 0.51 (increase) mg/g dw with DFO (n = 11), -1.27 DFP (n = 6), – 2.52 Comb (n = 45), and -1.30 with DFX (n = 16). These differences were significant between the DFO and Comb groups (p = 0.01).

• In the mild group with DFO the annual rate of change in LIC was 0.36 mg/g dw (n = 20), DFP 0.42 (n = 5), Comb -1.00 (n = 29), and DFX -0.19 (n = 16) (underlined result indicate increase in LIC). These differences were significant only between the DFO and Comb groups (p = 0.004).

There were no significant differences in the severe group.

Table 4 shows the number of patients who at some time had an acceptable level of hepatic T2* (≥ 9 ms) and whose T2* fell below that level. Comb had significantly fewer falls below the acceptable level than the other three regimes. The risk of the hepatic T2* falling below 9 ms (>3 mg/g dw) was higher in patients taking DFO, DFX and DFP compared to those on Comb (logistic regression analysis): DFO vs Comb: OR = 8 (p = 0.029); DFX vs Comb: OR = 24 (p = 0.001); DFP vs Comb OR = 16 (p = 0.002). All other comparisons were not significant. Table 5 shows the fall in LIC (mg/g dw) in those patients according to the regime they were receiving. The increase in LIC was greatest as follows: DFX>DFP>DFO>Comb (Kruskal Wallis test – result marginally non-significant p = 0.059).

**Table 4:** Number of patients whose hepatic T2* fell below 9 ms (>3 mg/gdw) according to chelation regime

	Medication				
	
	DFP	Comb	DFX	DFO	Total
Fall < 9, n (%)					

Unchanged, n (%)	7 (50)	32 (94)	4 (40)	8 (66.7)	51 (73)

Total, n (%)	14 (100)	34 (100)	10 (100)	12 (100)	70 (100)

Fisher's exact: p < 0.001

**Table 5:** Mean increase in hepatic iron in mg/gm/dry weight in patients who had an acceptable level of hepatic iron and who then fell below that level, depending on their chelation regime.

Treatment	Increase in hepatic iron		
	
	n	Mean	Range
DFO	4	3.5	0.6–10.8

DFP	7	4.6	1.9–13.2

Comb	2	0.8	0.6–1.0

DFX	6	7.3	1.2–15.5

Kruskal-Wallis test if means differ among groups: p = 0.059

## Discussion

The knowledge of the degree of organ specific iron loading and the ability to tailor iron chelation regimes are significant factors in the improved survival recently reported [4]. The aim of this study was to evaluate effects of iron chelators in a clinical setting rather than in controlled clinical trials. The outcomes confirm both findings from other studies and observations that have been seen in clinical practice.

One of the advantages of this study is that it is from a single large thalassaemia unit, which practices relatively consistent management. To date, there have been no reports on longitudinal measurements of iron load assessment with T2* that have analysed the efficacy of all four currently available chelation regimes. The only available report that analyzed repeated measurements assessed other parameters, not the rates of change or the efficacy of the available chelators [19]. The large number of patients followed with repeated studies allows the representation of time trends that might be expected according to the chelation regime prescribed. The type of analysis used demonstrated that the rate of change in T2* in the heart and liver varies according to the degree of baseline iron loading.

With respect to the heart, the lack of significant reduction in iron by standard treatment with DFO (30–40 mg/kg/infusion 5–6 days per week) in the mild and moderate iron loaded patients could be attributed to the relatively small sample sizes in the stratified analysis ("Initial interval analysis") or poor compliance. In the heavily loaded group the improvement was significant. This discrepancy may be related to better compliance in those patients who were concerned about their cardiac iron and to higher dosage and frequency of use of DFO. This result is consistent with those in patients with heavy cardiac iron load and cardiac dysfunction who were treated with intensive regimes with DFO continuously intravenously [20]. However in the overall analysis the results are derived from 32 patients and the lack of efficacy is in accordance with the incidence of cardiac related deaths and the onset of *de novo *cardiac disease in patients on DFO [21,22]. These observations are also consistent with studies that showed that despite DFO treatment, between 56 and 65% of patients had cardiac iron loading and 11–25% had excessive loading [8-10]. The results with DFP showing a significant rate of improvement in cardiac iron conform with the Greek and Italian randomised controlled study which showed superiority of DFP over DFO in its ability to improve T2* [18]. They also support findings of the lower incidence of cardiac deaths and de novo cardiac events from the two abovementioned Italian Studies [21,22].

Both a randomised placebo controlled study and a non-randomised study from Sardinia showed that the most rapid clearance of cardiac iron could be effected by Comb, consistent with this study [23,24]. They are also consistent with at least three case reports that demonstrated the reversal of cardiac failure with the use of combination therapy [25-27]. For a similar reversal with DFO, intensification of the therapy is necessary, usually by continuous intravenous infusions [20,28,29]. No patients in our study were on such intensive therapy. If such therapy is considered necessary, Comb is chosen by most patients, provided they do not have any contraindication to that therapy.

As we did not assess compliance, this study demonstrates relatively realistic outcomes that can be expected in a clinical setting. It is likely that poorly compliant patients may have negatively influenced outcomes but this confirms the value of this type of study compared to a strict clinical trial. The one patient who showed an increase in cardiac iron load most likely did not accept the therapy prescribed. The same patient demonstrated a further deterioration on her subsequent MR, as both liver and heart iron increased, consistent with poor compliance.

As yet, there are no randomized prospective studies with respect to the comparison of DFX with other regimes in their effect on cardiac iron. One prospective study has shown a significant reduction in cardiac iron with DFX over a 12 months period, that also included a stratification of patients using T2* <10 ms (7.4 – 8.2 ms geometric mean difference with p = 0.0002) as cut off for severe iron loading and between 10–20 ms for mild to moderate (14.6–17.4 ms p < 0.0001). This study is from a subset of patients who were participating in a clinical trial (EPIC) and the mean dose of DFX was 32.6 mg/kg/day with only 4.4% receiving less than 30 mg/kg/day [30]. In addition, two series of observations have demonstrated some benefit with increased cardiac T2* [31,32]. Over a longer period of observation, our results did not show a statistically significant difference in cardiac iron load in patients on DFX which are similar to the findings of MR observations after 4 years for patients entered into preregistration and extension phases of DFX studies [33] which showed that among patients with baseline T2* values < 20 ms, 7 improved, of whom 2 patients normalised T2*, 3 remained stable (change ≤ 0.2 ms) and 3 showed T2* deterioration. Similarly, a large Italian multicentre observational study showed that patients on DFX had greater cardiac iron load than those on DFO and DFP [34] after at least 12 months on the respective therapies. The latter report lacks baseline measurements of ferritin or T2* and for this reason the lower T2* with DFX may be related to higher initial total iron load. This may mean that patients with greater iron load were prescribed DFX preferentially. The difference between our observation and the Italian observations [34] compared to the two abovementioned prospective studies is most likely due to higher doses of drug (30 mg/kg/day with the ability to escalate the dose to over 40 mg/kg/day) used in those studies. The significance of compliance and dose was also stressed in the clinical follow up study [33]. In Europe DFX is not yet licensed above 30 mg/kg/day. Further randomised studies to compare DFX to other regimes in removing cardiac iron are anticipated.

The results derived from the "Initial interval analysis" confirm to a certain extent the overall conclusions that the repeated measurements produced. In some cases the applied stratification reduced the ability to distinguish statistically significant differences.

With respect to the liver, we did not use R2 for LIC determination [35] as our MR laboratory reports results to us using the T2* method. As was the case in this study, the lack of significant change in the LIC with DFO [36] and DFP [18] has been demonstrated in the past, though in general they have both been reported to reduce tissue iron [37,38]. The result with DFP reflects the small number of patients on that regime whose T2* was below the acceptable levels of hepatic iron and for this reason power for detecting statistical significance was reduced. The benefit of Comb with respect to its significant reduction in hepatic iron conforms to the abovementioned Sardinian study [23]. With respect to DFX, the "Initial interval analysis" with the first two MR scans showed a significant improvement in hepatic iron load at severe and moderate loads, similar to the findings in a large clinical trial [38]. The increases observed in patients who initially had LIC lower than 3 mg/gdw is difficult to explain. They may be related to reduced compliance, in that patients may have a false sense of security when they believe that their liver iron is low or because the dose prescribed was reduced. These outcomes should be taken into consideration if changes in dosage of DFX are being considered.

Considering the risk of LIC increasing with DFX, DFP and DFO in patients who initially had an acceptable LIC frequent monitoring with MR in patients receiving monotherapy is recommended. This is essential in cases in which they have been changed to monotherapy after they had received Comb with effective clearance of iron.

Taking into account that negative iron balance with the usual chelation regimes (dose and frequency) can only be achieved in all patients with the use of Comb [39] its superiority in maintaining patients at acceptable levels is understandable.

The drawbacks of our study were that it was not prospective, the prescription of a particular regime was made on clinical grounds and not on randomisation, the time period between MR scans was variable, we did not take into account whether the patients were splenectomised nor assess the annual red cell consumption and rate of iron loading. The latter has been shown to be important with respect to choice of dose of chelators [39,40]. In addition, patients on DFO, DFP and Comb were on those regimes for significantly longer than patients who received DFX. The resulting potential selection bias was diminished by stratified analysis which allowed comparison of patients with similar baseline characteristics. The problem with the variable time intervals was taken into account by incorporating time into the repeated measurements analysis and assessing rates of change with the "Initial interval analysis", including the restriction that a minimum of twelve months between the studies was necessary. The latter approach in the "Initial interval analysis" allowed sufficient time for each regime to demonstrate its capabilities and the usage of rates of change allowed for comparison of the data. In addition, we did not assess compliance nor make the analyses according to the doses of medications. In several instances the patients only had T2* of the heart measured thus explaining the smaller number of observations on the liver. This only rendered the performance of the repeated measurement analysis for the liver inappropriate, but did not have any effect on the "Initial interval analysis". In some cases the small number of patients in some sub-groups may have reduced the power to detect significant changes. The use of CIC was derived from a formula that was based on experimental data and not on calibration with human cardiac tissue. However the CIC results are compatible with the autopsy findings in four patients who died of cardiac iron loading during the 1970's [41]. More specifically the CIC in the hearts of those patients after being divided into smaller slices for analysis was heterogeneously between 0.49 and 2.0% dry weight (equivalent to 4.9 and 20 mg/gdw which according to the formula we used is equivalent to T2* 4.7 and 1.3 ms respectively).

The mean baseline cardiac T2* was greatest in patients on DFX. This reflects current practice in our unit in that we tended to recommend this regime to patients whom we knew did not have excessive cardiac iron. Conversely, patients who were taking DFP had the highest hepatic T2* levels (low LIC), which is consistent with our clinical practice in that DFP is not our first choice when aiming to achieve a significant reduction in hepatic iron. It can be seen that the largest group of patients were those taking Comb. The explanation for this is that prior to 1999, practically all of our patients were exposed only to DFO and many patients had excessive cardiac iron. As it was becoming recognised that the most effective regime for reducing cardiac iron was use of Comb [42], this regime was recommended to many patients.

## Conclusion

The management of transfusion dependent thalassaemia has been revolutionised over the last 10 – 15 years. This is a result of both the availability of better and non-invasive methods of accurately assessing iron load in the heart and liver and the option of tailoring chelation according to the degrees of iron load using any of the three chelators or combinations thereof. Comb therapy is best in reducing both cardiac and hepatic iron, while DFP and DFX monotherapies are effective in the heart and liver respectively. If reduction of iron load is regarded as being relatively urgent, combining DFP and DFO is the most appropriate option, provided the patients have no contraindication to such therapy. All these results represent findings that can be expected in the real life clinical setting compared to clinical trials. Desirable options for future research include testing the efficacy of combinations of DFO/DFX and DFP/DFX. The treatment of no other formerly fatal genetic disease has progressed as far as that of thalassaemia. With current advances, survival and well-being should continue to improve even more.

## Competing interests

VB is a remunerated consultant with ApoPharma Inc and a consultant with Novartis Inc with respect to the development of desferasirox, has received honoraria from Demo AE (Greece) and served on the Advisory Board of ApoPharma and has attended Novartis sponsored meetings on iron chelation.

GC, PG, KZ have no interests to declare.

EB and VL have been remunerated for clinical trials from both ApoPharma Inc and Novartis Inc and have attended sponsored meetings from both. VL has also served on an ApoPharma advisory board.

## Authors' contributions

All authors were involved in determining the design of the trial and all except for VL were involved in the data entry. GC performed the statistical analysis. The paper was written by VB, GC and VL with contributions from the other authors.

## References

[B1] Ladis V, Chouliaras G, Berdousi H, Kanavakis E, Kattamis C (2005). Longitudinal study of survival and causes of death in patients with thalassemia major in Greece. Ann N Y Acad Sci.

[B2] Borgna-Pignatti C, Rugolotto S, De Stefano P, Zhao H, Cappellini MD, Del Vecchio GC, Romeo MA, Forni GL, Gamberini MR, Ghilardi R (2004). Survival and complications in patients with thalassemia major treated with transfusion and deferoxamine. Haematologica.

[B3] Modell B, Khan M, Darlison M (2000). Survival in beta-thalassaemia major in the UK: data from the UK Thalassaemia Register. Lancet.

[B4] Modell B, Khan M, Darlison M, Westwood MA, Ingram D, Pennell DJ (2008). Improved survival of thalassaemia major in the UK and relation to T2* cardiovascular magnetic resonance. J Cardiovasc Magn Reson.

[B5] Caro JJ, Ward A, Green TC, Huybrechts K, Arana A, Wait S, Eleftheriou A (2002). Impact of thalassemia major on patients and their families. Acta Haematol.

[B6] Aessopos A, Farmakis D, Hatziliami A, Fragodimitri C, Karabatsos F, Joussef J, Mitilineou E, Diamanti-Kandaraki E, Meletis J, Karagiorga M (2004). Cardiac status in well-treated patients with thalassemia major. Eur J Haematol.

[B7] Berdoukas V, Dakin C, Freeman A, Fraser I, Aessopos A, Bohane T (2005). Lack of correlation between iron overload cardiac dysfunction and needle liver biopsy iron concentration. Haematologica.

[B8] Tanner MA, Galanello R, Dessi C, Westwood MA, Smith GC, Nair SV, Anderson LJ, Walker JM, Pennell DJ (2006). Myocardial iron loading in patients with thalassemia major on deferoxamine chelation. J Cardiovasc Magn Reson.

[B9] Aessopos A, Fragodimitri C, Karabatsos F, Hatziliami A, Yousef J, Giakoumis A, Dokou A, Gotsis ED, Berdoukas V, Karagiorga M (2007). Cardiac magnetic resonance imaging R2* assessments and analysis of historical parameters in patients with transfusion-dependent thalassemia. Haematologica.

[B10] Wood JC, Tyszka JM, Carson S, Nelson MD, Coates TD (2004). Myocardial iron loading in transfusion-dependent thalassemia and sickle cell disease. Blood.

[B11] Angelucci E, Brittenham GM, McLaren CE, Ripalti M, Baronciani D, Giardini C, Galimberti M, Polchi P, Lucarelli G (2000). Hepatic iron concentration and total body iron stores in thalassemia major. N Engl J Med.

[B12] Borgna-Pignatti C, Vergine G, Lombardo T, Cappellini MD, Cianciulli P, Maggio A, Renda D, Lai ME, Mandas A, Forni G (2004). Hepatocellular carcinoma in the thalassaemia syndromes. Br J Haematol.

[B13] Anderson LJ, Holden S, Davis B, Prescott E, Charrier CC, Bunce NH, Firmin DN, Wonke B, Porter J, Walker JM, Pennell DJ (2001). Cardiovascular T2-star (T2*) magnetic resonance for the early diagnosis of myocardial iron overload. Eur Heart J.

[B14] Kattamis A, Ladis V, Berdousi H, Kelekis NL, Alexopoulou E, Papasotiriou I, Drakaki K, Kaloumenou I, Galani A, Kattamis C (2006). Iron chelation treatment with combined therapy with deferiprone and deferioxamine: a 12-month trial. Blood Cells Mol Dis.

[B15] Westwood MA, Firmin DN, Gildo M, Renzo G, Stathis G, Markissia K, Vasili B, Pennell DJ (2005). Intercentre reproducibility of magnetic resonance T2* measurements of myocardial iron in thalassaemia. Int J Cardiovasc Imaging.

[B16] Wood JC, Otto-Duessel M, Aguilar M, Nick H, Nelson MD, Coates TD, Pollack H, Moats R (2005). Cardiac iron determines cardiac T2*, T2, and T1 in the gerbil model of iron cardiomyopathy. Circulation.

[B17] Wood JC, Enriquez C, Ghugre N, Tyzka JM, Carson S, Nelson MD, Coates TD (2005). MRI R2 and R2* mapping accurately estimates hepatic iron concentration in transfusion-dependent thalassemia and sickle cell disease patients. Blood.

[B18] Pennell DJ, Berdoukas V, Karagiorga M, Ladis V, Piga A, Aessopos A, Gotsis ED, Tanner MA, Smith GC, Westwood MA (2006). Randomized controlled trial of deferiprone or deferoxamine in beta-thalassemia major patients with asymptomatic myocardial siderosis. Blood.

[B19] Noetzli LJ, Carson SM, Nord AS, Coates TD, Wood JC (2008). Longitudinal analysis of heart and liver iron in thalassemia major. Blood.

[B20] Anderson LJ, Westwood MA, Holden S, Davis B, Prescott E, Wonke B, Porter JB, Walker JM, Pennell DJ (2004). Myocardial iron clearance during reversal of siderotic cardiomyopathy with intravenous desferrioxamine: a prospective study using T2* cardiovascular magnetic resonance. Br J Haematol.

[B21] Borgna-Pignatti C, Cappellini MD, De Stefano P, Del Vecchio GC, Forni GL, Gamberini MR, Ghilardi R, Piga A, Romeo MA, Zhao H, Cnaan A (2006). Cardiac morbidity and mortality in deferoxamine- or deferiprone-treated patients with thalassemia major. Blood.

[B22] Piga A, Gaglioti C, Fogliacco E, Tricta F (2003). Comparative effects of deferiprone and deferoxamine on survival and cardiac disease in patients with thalassemia major: a retrospective analysis. Haematologica.

[B23] Tanner MA, Galanello R, Dessi C, Smith GC, Westwood MA, Agus A, Roughton M, Assomull R, Nair SV, Walker JM, Pennell DJ (2007). A randomized, placebo-controlled, double-blind trial of the effect of combined therapy with deferoxamine and deferiprone on myocardial iron in thalassemia major using cardiovascular magnetic resonance. Circulation.

[B24] Tanner MA, Galanello R, Dessi C, Smith GC, Westwood MA, Agus A, Pibiri M, Nair SV, Walker JM, Pennell DJ (2008). Combined chelation therapy in thalassemia major for the treatment of severe myocardial siderosis with left ventricular dysfunction. J Cardiovasc Magn Reson.

[B25] Wu KH, Chang JS, Tsai CH, Peng CT (2004). Combined therapy with deferiprone and desferrioxamine successfully regresses severe heart failure in patients with beta-thalassemia major. Ann Hematol.

[B26] Tsironi M, Deftereos S, Andriopoulos P, Farmakis D, Meletis J, Aessopos A (2005). Reversal of heart failure in thalassemia major by combined chelation therapy: a case report. Eur J Haematol.

[B27] Tavecchia L, Masera N, Russo P, Ciro A, Vincenzi A, Vimercati C, Masera G (2006). Successful recovery of acute hemosiderotic heart failure in beta-thalassemia major treated with a combined regimen of desferrioxamine and deferiprone. Haematologica.

[B28] Davis BA, Porter JB (2000). Long-term outcome of continuous 24-hour deferoxamine infusion via indwelling intravenous catheters in high-risk beta-thalassemia. Blood.

[B29] Miskin H, Yaniv I, Berant M, Hershko C, Tamary H (2003). Reversal of cardiac complications in thalassemia major by long-term intermittent daily intensive iron chelation. Eur J Haematol.

[B30] Pennell DJ, Porter JB, Cappellini MD, Li C-K, Aydinok Y, Chan LL, Kattamis A, Smith G, Habr D, Domokos G, Hmissi A, Taher A (2008). Efficacy and safety of deferasirox (Exjade) in reducing cardiac iron in patients with β-thalassemia major: Results from the cardiac substudy of the EPIC trial. ASH Annual Meeting, San Francisco, USA, 6–9 December.

[B31] Eleftheriou P, Tanner M, Pennell D, Porter J (2006). Response of myocardial T2* to oral deferasirox monotherapy for 1 year in 29 patients with transfusion-dependent anaemias; A subgroup analysis. Haematologica.

[B32] Wood J, Thompson A, Paley C, Kang B, Giardina P, Harmatz P, Virkus J, Coates T (2008). Deferasirox (Exjade) monotherapy significantly reduces cardiac iron burden in chronically transfused β-thalassemia patients: An MRI T2* study. ASH Annual Meeting, San Francisco, USA, 6–9 December.

[B33] Garbowski M, Eleftheriou P, Pennell D, Tanner M, Porter JB (2008). Impact of Compliance, Ferritin and LIC on Long-Term Trends in Myocardial T2* with Deferasirox. ASH Annual Meeting.

[B34] Pepe A, Favilli B, Positano V, Cianciulli P, Spasiano A, Capra M, Borgna-Pigniatti C, Putti MC, Casini T, Romeo MA, Bisconte MG, Filosa A, Caruso V, Quarta A, Pitrolo L, De Sanctis V, Gerardi C, Maggio C, Pietrangelo A, Lombardi M (2008). Comparison of deferasirox, deferiprone and desferrioxamine effectiveness on myocardial iron concenrations and biventricular function by quantitative MRI in thalassemia major. ASH Annual Meeting, San Francisco, USA, 6–9 December.

[B35] St Pierre TG, Clark PR, Chua-Anusorn W (2005). Measurement and mapping of liver iron concentrations using magnetic resonance imaging. Ann N Y Acad Sci.

[B36] Berdoukas V, Bohane T, Tobias V, De Silva K, Fraser I, Aessopos A, Lindeman R (2005). Liver iron concentration and fibrosis in a cohort of transfusion-dependent patients on long-term desferrioxamine therapy. Hematol J.

[B37] Wu SF, Peng CT, Wu KH, Tsai CH (2006). Liver fibrosis and iron levels during long-term deferiprone treatment of thalassemia major patients. Hemoglobin.

[B38] Cappellini MD, Cohen A, Piga A, Bejaoui M, Perrotta S, Agaoglu L, Aydinok Y, Kattamis A, Kilinc Y, Porter J (2006). A phase 3 study of deferasirox (ICL670), a once-daily oral iron chelator, in patients with beta-thalassemia. Blood.

[B39] Grady R, Berdoukas V, Rachmielewitz EA, Giardina PJ (2000). Optimising chelation therapy: Combining deferiprone and deferoxamine. Blood.

[B40] Cohen AR, Glimm E, Porter JB (2008). Effect of transfusional iron intake on response to chelation therapy in beta-thalassemia major. Blood.

[B41] Modell B, Berdoukas V (1984). The Clinical Approach to Thalassaemia.

[B42] Wonke B, Wright C, Hoffbrand AV (1998). Combined therapy with deferiprone and desferrioxamine. Br J Haematol.

